# Study the Effect of Relative Energy Deficiency on Physiological and Physical Variables in Professional Women Athletes: A Randomized Controlled Trial

**DOI:** 10.3390/metabo13020168

**Published:** 2023-01-23

**Authors:** Laura Miralles-Amorós, Nuria Asencio-Mas, María Martínez-Olcina, Manuel Vicente-Martínez, José Manuel García-De Frutos, Marcelo Peñaranda-Moraga, Lucía Gonzálvez-Alvarado, Rodrigo Yáñez-Sepúlveda, Guillermo Cortés-Roco, Alejandro Martínez-Rodríguez

**Affiliations:** 1 Department of Analytical Chemistry, Nutrition and Food Science, University of Alicante, 03690 Alicante, Spain; 2 Faculty of Health Science, Miguel de Cervantes European University, 47012 Valladolid, Spain; 3 Physical Activity and Sport Sciences Department, Faculty of Sport, Catholic University San Antonio of Murcia, 30107 Murcia, Spain; 4 Faculty of Education and Social Sciences, Universidad Andres Bello, Viña del Mar 2520000, Chile; 5 Escuela de Educación, Pedagogía en Educación Física, Entrenador Deportivo, Universidad Viña del Mar, Viña del Mar 2520000, Chile; 6 Alicante Institute for Health and Biomedical Research (ISABIAL), 03010 Alicante, Spain

**Keywords:** energy deficit syndrome, low energy availability, handball, rest metabolic rate, nutrition, performance, strength, eating disorders, body image, mood state

## Abstract

Energy deficits are often observed in athletes, especially in female athletes, due to the high expenditure of sport and strict diets. Low energy availability can cause serious health problems and affect sport performance. The aim of this study was to evaluate the effects of different personalized dietary plans on physiological and physical factors related to energy deficit syndrome in female professional handball players. Twenty-one professional female handball players, aged 22 ± 4 years, 172.0 ± 5.4 cm and 68.4 ± 6.7 kg, divided into three groups (FD: free diet; MD: Mediterranean diet; and AD: high antioxidant diet), participated in this 12-week randomized controlled trial. Energy expenditure through indirect calorimetry, energy availability, 7 day dietary intake analysis, blood pressure, cholesterol, menstrual function, body composition by both anthropometry and bioelectrical impedance, and strength performance were assessed. All participants showed low energy availability (<30 kcal/lean mass per day); despite this, all had eumenorrhea. Significant improvements were found after the intervention in all components of body composition (*p* < 0.05). In the remaining variables, despite slight improvements, none were significant neither over time nor between the different groups. Low energy availability has been observed in all professional female handball players, which may lead to serious consequences. A longer period of intervention is required to assess the differences between diets and improvements in other parameters.

## 1. Introduction

Energy availability (EA) is defined as the amount of dietary energy available to maintain physiological function after subtracting energy expenditure from physical activity, or as the difference between energy intake (EI) and energy expenditure during exercise (EEE), which also takes into account an individual’s lean body mass [1,2]. Previous research has set a benchmark of 30 Kcal/kg lean body mass/day, beyond which a lower figure results in a disruption of the normal hormonal environment [2].

Relative Energy Deficiency Syndrome (RED-S) was recognized by the International Olympic Committee (IOC) in 2014 as an insufficient caloric intake, and/or excessive energy expenditure [3]. This syndrome is characterized by low energy availability (LEA), sufficient for body functions involved in optimal athlete health and performance are not adequately produced [4]. It is for this reason that the main etiological factor compromising health status is LEA, which results in long-term and potentially irreversible damage to athletes’ health [5,6].

LEA has been correlated with a decreased Resting Metabolic Rate (RMR) in female endurance athletes [4,7]. Prospectively, increasing the training load in athletes translates into a significant reduction in RMR [8]; however, an improved metabolism has been observed with modest increases in body weight and body fat percentage [9]. In addition, LEA may promote susceptibility to respiratory tract infections and negatively affect blood lipid levels [10].

Maintaining dietary restriction over a prolonged period appears to negatively affect athletic performance through the depletion of glycogen stores, interference with protein synthesis, or impeding quality training due to an increased risk in injury and illness [11,12,13]. This leads to a premature reduction in physical, psychological, and mental capacity [14,15]. Some research has explored the effect of RED-S on aerobic performance [4,16]; however, no publication has examined its effect on strength performance [10].

Studies have shown that the intake of female athletes is often insufficient to meet nutritional and energy needs (both macronutrients and micronutrients). Increasingly, a higher percentage of athletes competing in non-weight related sports are at increased risk of LEA [17,18]. Handball is a complex Olympic discipline characterized by fast, high intensity movements with the aim of scoring goals. The high physical requirements of this sport require players to have optimal anthropometric qualities [19].

Since the publication of the IOC consensus documents [4,5], research on RED-S and LEA has increased, and the prevalence of LEA is of concern. Recent studies have shown that 45% of female recreational exercisers were at risk of LEA [20], while another study found a high prevalence of LEA in both male and female elite youth athletes (males, 56%; females, 51%) [6,21].

The basis of RED-S is an energy deficiency; therefore, one of the main pillars of treatment should focus on increasing calorie intake and/or decreasing calorie expenditure (i.e., from training) to correct the energy imbalance. Existing evidence suggests that a diet rich in antioxidants and anti-inflammatory foods, and exercise planning, improves cardiovascular response, performance, and post-exercise recovery [13].

The Mediterranean Diet is valued for the wide range of benefits it provides, as it is characterized by a high content of essential nutrients and micronutrients. Several studies have shown that these nutrients have an important chemical-preventive role on oxidative stress [22]. On the other hand, one food group with a high anthocyanin content is red fruits. These have polyphenols that have been associated with improved cardiovascular risk profiles, and a decrease in comorbidities [23]. Human intervention studies using different versions of berries have shown significant improvements in low-density lipoprotein oxidation, lipid peroxidation, total plasma antioxidant capacity, dyslipidemia, and reduced levels of molecular biomarkers of cardiovascular disease [23].

LEA can predict markers of suboptimal health and functioning [24,25,26], a rigorous assessment of EA can serve as a diagnostic tool in RED-S prevention or management. Laboratory trials in women have shown that 45 kcal/kg lean body mass/day is required for EA with healthy physiological functions [4]. Screening and low-risk EA interventions are needed to protect the health and performance of athletes [10].

The review of the scientific literature shows that LEA can be accompanied by physiological and physical disorders. The hypothesis of this research is that dietary and nutritional intervention specifically adapted and individualized to female handball players provides positive effects on body composition, health status, and sporting performance.

The aim of this study was to evaluate the effects of personalized dietary planning in professional female athletes on the physiological and physical effects related to RED-S. A secondary objective was to analyze the effect of Mediterranean diets and antioxidant diets on these factors.

## 2. Materials and Methods

### 2.1. Study Design

**Figure 1:**
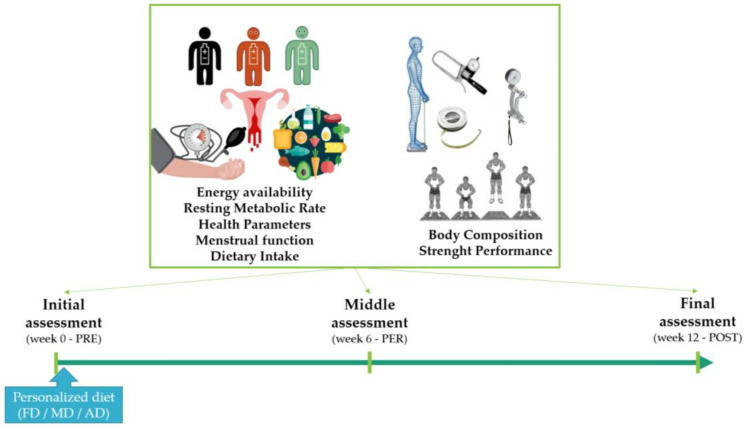
Study Design. F.D: free diet group; MD: Mediterranean diet experimental group; AD: antioxidant diet experimental group; PRE: before intervention; PER: during intervention; POST: after intervention.

A randomized controlled trial was conducted for 12 weeks (Figure 1). The study involved the best Spanish national players in this sport (winners of the Super Cup and Queen’s Cup), who represent elite handball players from all over the world.

The guidelines of the Declaration of Helsinki (revised in Fortaleza, Brazil, in October 2013), and the recommendations of Good Clinical Practice of the EEC (Document 111/3976/88 of July 1990) were followed in all the procedures conducted. Informed consents were completed by all participants prior to the beginning. The research was approved by the Ethics Committee of the University of Alicante (Spain) (UA-2021-03-11). In addition, the research was registered in the official clinical trials database, (ClinicalTrials.gov, accessed on 13 December 2022), obtaining the registration number NCT05567211. The investigators maintained the confidentiality of all participants’ personal data, coding personal information for this purpose.

### 2.2. Subjects

A total of 21 senior professional female handball players (Guerreras Iberdrola league), participated in the study. All of them performed 10 h to 15 h of training per week, interspersed with competitive events. The following exclusion criteria were considered: presence of chronic diseases, medication influencing the results, and refusal to sign the informed consent form. However, no female player refused to participate, and no athlete was excluded. There was no financial compensation to participants for their collaboration. The anonymity of the players was always preserved.

The subjects were randomly assigned electronically by block design into three arms (Free Diet, Mediterranean Diet, and Antioxidant Diet) using online software according to published recommendations [27]. A researcher who was not involved in the evaluations or interventions of this study prepared these envelopes.

### 2.3. Data Collection

#### 2.3.1. Physiological Variables

##### Energy Availability and Resting Metabolic Rate

RMR was measured by indirect calorimetry with the blueberry program (METALYZERR 3 B-R3 spirometer, Cortex Biophysik GmbH, Leipzig, Germany). According to the manufacturer’s instructions, prior to each test, gas exchange and ventilation were calibrated. High-resolution spiroergometric systems were transmitted by an amperometry solid electrolyte sensor for the O_2_ assessment and an infrared sensor for the CO_2_ assessment, which continuously recorded through breath-by-breath gas analysis. A small division of the breathable air through the volume flow sensor was performed using an ergonomic mask. RMR was assessed by measuring the amount of O_2_ consumed and CO_2_ produced. RMR was assessed in the morning after a night of restful sleep, and after a night of fasting. Participants were asked to avoid intense exercise and alcohol or caffeine consumption for one day prior to RMR assessment. RMR was measured for 30 min in a quiet room and in a stable position. Respiratory exchange ratio and oxygen consumption (VO_2_) were investigated within the average 20 min of the predicted rest period. RMR was determined using the Harris–Benedict equation, which considers the age, weight, and height of the individuals. In addition, the EA was measured by means of the formula: ⦏Energy intake (EI) (Kcal) −Exercise energy expenditure (EEE) (kcal)⦐/Lean body mass (Kg).

The Low Energy Availability in Women Questionnaire (LEAF-Q) is a tool used for early detection of RED-S before significant changes in bone mineral density (BMD) and body composition occur. The questionnaire examines physiological symptoms reported by female athletes related to LEA, and includes sequential questions on injury, gastrointestinal, and reproductive functions. It has been validated in endurance athletes and has a specificity of 90% and a sensitivity of 78%. A score of ≥8 out of 25 questions indicates that the athlete is at risk of LEA and, as a consequence, may be at risk of developing the RED-S symptom cluster [28].

##### Health Parameters

Blood pressure

Blood pressure (systolic and diastolic blood pressure) was measured with participants seated and relaxed using an Omron M4 Intelli IT blood pressure monitor (WendenstraBe, Hamburg, Germany).

Cholesterol

Cholesterol (mg/dL), in drip loss were determined by the strip method using an Accutrend Plus apparatus (Roche Diagnostic GMBH, Mannheim, Germany). The tests were conducted using dedicated reactive strips. The test result was obtained at: 168 s after placing a 20 µL (microliters) drop on a corresponding reagent strip.

Menstrual cycle

Regarding menstrual function, all measurements were taken at the same time of the cycle, which is the mid-luteal phase (between days 19 and 22 of the cycle). Measurements were taken at this phase because numerous reviews and meta-analyses have indicated that this was the phase in which strength conditioning was enhanced [29,30].

In the previous months, a record of the menstrual cycles was made through validated applications [31], and physiological alterations that could be observed during the periods (changes in body temperature, irritability, increased appetite, etc.) in order to monitor the different phases of the cycle and be able to carry out the measurements of the research at the appropriate time.

##### Dietary Intake

Using a dietary record administered with the help of the Dietopro.com program [32], both qualitative and quantitative assessments were made of the food consumed in a week at the three points in time of the study. Regarding the quantitative analysis, the dietary records obtained for 1 week at each measurement time were entered into Dietopro.com software to obtain information on energy, macronutrients (carbohydrates, proteins, lipids, and fiber), and micronutrients (vitamins and minerals) consumed during the different periods. Players were trained prior to self-completion of the questionnaire to quantify all foods and beverages consumed, provided with photos and instructional videos to promote accurate self-reporting, without overlooking any ingredient, and with information on food weighing and quantity consumed. Daily intakes collected over 7 consecutive days at the three points in time were averaged, obtaining both absolute values as a percentage and per kg of weight per day.

#### 2.3.2. Physical Variables

##### Body Composition

Anthropometric variables were measured for each subject. For this purpose, the restricted profile was developed, following the standard protocol of the International Society for the Advancement of Kinanthropometry (ISAK) [33].

All measurements were performed by the same investigator, an ISAK level 2 anthropometrist. The mean technical error was less than 1% for perimeters, circumferences, lengths and heights, and less than 5% for skinfolds. All measurements were performed under the same conditions, early in the day, under fasting and basal conditions, with the same material and at room temperature (22 ± 1 °C).

Height and sitting height were determined with the participant’s head held in the position of the horizontal Frankfort plane. Length of the wingspan; eight skinfolds (subscapular, tricipital, bicipital, iliac crest, supraspinal, abdominal, anterior thigh, and medial calf); six perimeters (relaxed arm, contracted arm, waist, hip, mid-thigh, and leg); three bone diameters (humerus biepicondylar, femur epicondylar, and wrist bistiloid) were collected. The sum of six skinfolds (subscapular, triceps, supraspinal, abdominal, anterior thigh, and medial calf) and eight skinfolds (all included) were also calculated.

Height and sitting height were determined using a mobile anthropometer (Seca 213, SECA Deutschland, Hamburg, Germany) to the nearest millimeter. For the measurement of perimeters, a 2 m narrow, inextensible metal tape measure (Lufkin, TX, USA; accuracy, 1 mm); a small bone diameter pachymeter; a skinfold caliper (Harpenden, UK; accuracy, 0.2 mm) was used; weight was recorded with a digital scale (BC545N, Tanita, Tokyo, Japan; accuracy, 100 g); and supplementary material (demographic pencil for marking players) and anthropometric bench 40 × 50 × 30 cm.

Body composition was calculated using the 4-component model: muscle mass, fat mass, bone mass, and residual mass. Fat mass was estimated using the formula of Withers et al. [34], Carter [35], and Faulkner [36]. Muscle mass was determined using the formula of Lee et al. [37], and bone mass was determined using the formula of Rocha [38]. According to the Spanish Committee of Kinanthropometry, these methods are the most suitable for high-performance players [38].

The somatotype is defined as the quantification of the shape and composition of the human body. It is represented by three components: (1) endomorphy, (2) mesomorphy, and (3) ectomorphy. The mean somatotype and classification were determined using the anthropometric method of Heath and Carter [39] and their classification. Each component was calculated with its corresponding formula [40].

Furthermore, the dual indirect bioelectrical impedance method was used to determine body composition and hence nutritional status using a multi-frequency, multi-algorithm, French-made, European Community quality-endorsed, portable, painless, and cordless BiodyXpert (CE MEDICAL, France). It has direct hand/foot measurement with sensors integrated in the device (no cable electrodes), weighing only 300 g. More than 77 biomarkers/data with a single measurement [41].

##### Strength Performance

Countermovement Jump Test

In the countermovement jumping test (CMJ), participants performed a maximal vertical jump from a standing position, without allowing the arms to swing and with a knee flexion of 90°. Several familiarization tests were performed prior to the test [42]. A contact platform (Optojump Next Microgate, Bolzano, Italy) was used to measure the CMJ. Three measurements were performed with 30 s of recovery in between. The flight time was used to calculate the jump height. The best jump was used for subsequent analysis [43,44].

Abalakov Jump Test

The Abalakov Jump Test was used. Participants performed three countermovement’s with 30 s rest between jumps [45] performed on a stable surface. All players had to stand up and perform a 90° knee flexion followed by the fastest possible extension, aiming to reach the highest possible jump height. As in CMJ test, an infrared optical data collection system (Optojump Next Microgate, Bolzano, Italy) was used to calculate the height of the Abalakov jump. Of the three results, the best one was used for statistical analysis.

Handgrip Test

A Jamar handheld hydraulic dynamometer was used with the arm at right angles and the elbows at the sides of the body. The instrument was adjusted, its base rested on the first metacarpal and the handle rested on the middle of the participant’s four fingers. All players performed the test with their dominant and non-dominant hand. Their maximal isometric effort was maintained for 5 s. The test was performed twice, with a 1 min rest between attempts, with the higher value being the one to be used in the subsequent analysis.

Performance tests were conducted mid-morning, 2 h after breakfast, with optimal hydration, rest, and after more than 36 h without physical activity.

### 2.4. Intervention

The intervention in the present study was based on the follow-up of a personalized dietary-nutritional plan for each of the players for 3 months (12 weeks). Three groups were established randomly: (1) control group (FD) followed a free diet, with healthy lifestyle recommendations for women athletes; (2) the experimental Mediterranean Diet (MD) group; and (3) experimental group high in antioxidants diet (AD). Each group consisted of seven players. The different plans were equally structured so as not to draw attention to which group each player belonged to. The software used in the elaboration of the diet was Dietopro (Dietopro, Valencia, Spain) [46].

The proposed diets offered an isocaloric intake in accordance with the recommendations for professional female handball players [47]. Individualized diets were calculated to provide energy according to the training program to meet the recommended daily requirements in the three groups. For this purpose, total energy expenditure was estimated from the RMR obtained for each player and multiplied by the EEE and the thermic effect of the food. Daily physical activities and handball specific EEE was estimated using the METs and their equations [48].

Macronutrients were adjusted to the guidelines for handball athletes [47]. A total of 6–8 g/kg/day of carbohydrate (50–55% total caloric value), 1.4–1.6 g/kg/day of protein (15–17% total caloric value), and 30–35% of the total caloric value for fat were provided. The difference between the interventions was the type of food recommended to achieve these needs and the micronutrient intake [47]. MD prioritized whole grains, fish and vegetables, providing 15 mg for α-tocopherol (100% of the recommended daily allowance (RDA)), 180 mg for ascorbic acid (200% of the RDA), and 900 μg (micrograms) for vitamin A (100% RDA) [49]. The AD was based primarily on antioxidant fruit (pomegranate, blueberries, raspberries, and beetroot), which provided 30 mg for α-tocopherol (200% of the RDA), 450 mg for ascorbic acid (500% of the RDA) and 1800 μg for vitamin A (200% of the RDA) [50]. The players were also provided with a list of food equivalents and foods to avoid. They were contacted weekly by telephone to improve compliance with the nutritional advice.

### 2.5. Statistical Analysis

Jamovi 1.1.3.0 software was used for statistical analyses. Descriptive statistics (mean ± standard deviation) were calculated for all variables. Normality distribution was tested using the Shapiro–Wilk test. For equality of variances, Levene’s test was performed, and repeated measures ANOVA analysis was applied to analyze the effects of the intervention on the assessments. For time-by-group interaction effects, partial eta squared effect sizes (η^2^) were calculated. If significant main effects were found, post hoc (Bonferroni and Tukey) tests were performed. In addition, to establish connections between study variables, Pearson’s correlation test was used on correlations to determine effect sizes (small: 0.10, medium: 0.30, and high: 0.50) [51,52], with 95% confidence intervals. The level of statistical significance was set at *p* < 0.05.

## 3. Results

**Figure 2:**
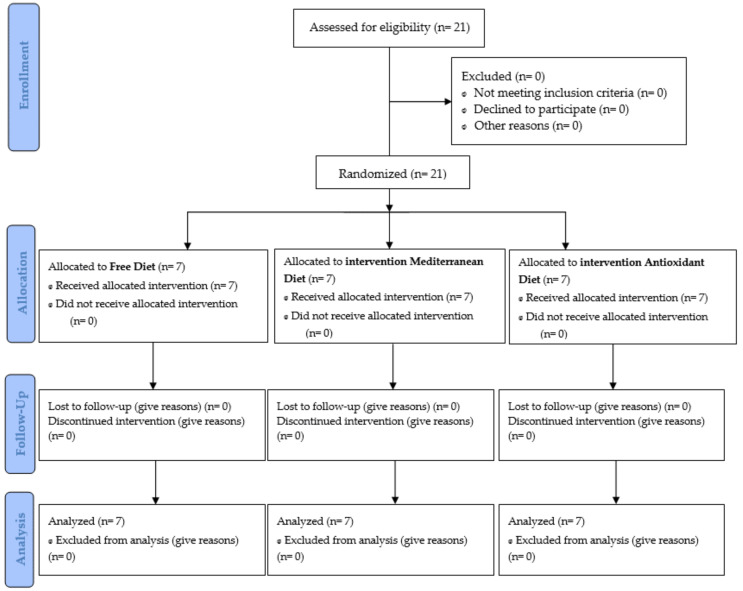
Consort Flow Diagram.

The study involved 21 professional female handball players aged 22 ± 4 years, 172.0 ± 5.4 cm, and 68.4 ± 6.7 kg, divided into three groups (FD: free diet; MD: Mediterranean diet; AD: high antioxidant diet) (Figure 2). Recruitment took place during the 2022–2023 pre-season. The intervention took place in August (PRE—0 weeks), during September-October (PER—6 weeks), November (POST—12 weeks) 2022.

From the score obtained in the LEAF-Q questionnaire, 13 of the female players were at risk of RED-S (61.9%), and all of them presented LEA (<30 kcal/kg lean body mass per day), showing no significant differences between groups *p* = 0.348 and *p* = 0.881, respectively. Regarding the possible confounding variables (height, weight, and BMI), no differences were observed between the groups. The mean and standard deviation of the data per group are shown in Table 1.

### 3.1. Physiological Variables

#### 3.1.1. Energy Availability and Resting Metabolic Rate

Regarding basal metabolism, energy availability, and LEAF-Q score, no significant differences were found either between groups, or over 12 weeks intervention (*p* > 0.05) (Table 2).

#### 3.1.2. Health Parameters

In terms of health parameters, blood pressure, pulse per minute, and cholesterol Table 3, no significant differences were found over time or between groups. All players had a regular and healthy menstrual cycle.

#### 3.1.3. Dietary Intake

Energy and nutritional intake showed no differences over time (Table 4). Significant differences were only shown in the percentage of vegetable protein between groups (*p* = 0.020) being higher at the end of the intervention than at the beginning. However, no significant differences were found by Bonferroni or Tukey’s post hoc analysis (*p* > 0.05).

As for the qualitative assessment, it can be said that the average number of intakes was five plus the meals related to training (during training and post-training). The quality of cooking appeared to be adequate, prioritizing grilling and baking techniques. Water intake was insufficient and, in general, there was no intake of dietary or sports supplements; the few who ingested them drank isotonic drinks during their sports practice. Finally, in terms of nutrient quality, most of the carbohydrates were slow-absorbing (potato and pasta), but there were also fast-absorbing carbohydrates, such as fruit and pastries. Proteins were mainly of high biological value (eggs, fish, and white meat). The main source of fat was extra virgin olive oil.

### 3.2. Physical Variables

#### 3.2.1. Body Composition

Table 5 and Table 6 show the body composition variables of the FD, MD, and AD groups in the three time periods measured from anthropometry and bioelectrical impedance, respectively. Statistically significant differences were observed in the variables weight (*p* = 0.030), 6-skinfold sum (*p* < 0.001), 8-skinfold sum (*p* < 0.001), percentage fat mass (*p* < 0.001), kilograms of fat mass (*p* < 0.001), percentage muscle mass (*p* < 0.001), kilograms of muscle mass (*p* < 0.001), percentage of bone mass (*p* = 0.030), kilograms of residual mass (*p* < 0.001), fat free dry mass (*p* = 0.020), protein mass (*p* = 0.010), metabolic protein mass (*p* = 0.010), and basal metabolism (*p* = 0.020) measured by bioelectrical impedance between the different time measures, but not between groups. Throughout the intervention, significant differences between groups were observed only in fat-free mass (*p* = 0.037), lean mass (*p* = 0.039), and total water (*p* = 0.024) measured by electrical bioelectrical impedance. However, after applying Bonferroni and Tukey post hoc analyses, no significant differences were observed. No significant differences were observed in bone mass and bone mineral content measured by both anthropometry and bioelectrical impedance.

#### 3.2.2. Strength Performance

For the strength performance tests (Table 7), significant differences were found over time for the Abalakov jump test, with jump height improving over time (*p* = 0.006). However, no differences were found in the CMJ jump and grip strength tests, either over time or between groups (*p* > 0.05).

### 3.3. Correlations

Statistically significant positive correlations were observed after the 12-week intervention between the variables Abalakov and CMJ (*p* < 0.001); non-dominant handgrip with the following variables: weight (*p* = 0.008), dominant handgrip (*p* = 0.007), muscle mass (*p* = 0.025), bone mass (*p* = 0.044), residual mass (*p* = 0.022), and fat-free mass by bioelectrical impedance (*p* = 0.005); LEAF-Q questionnaire score and energy intake (*p* = 0.002); protein intake per kilogram of body weight, and the next variables METs (*p* = 0.043), and energy availability and energy intake (*p* = 0.002); and skeletal muscle mass by bioelectrical impedance with non-dominant handgrip (*p* = 0.001).

Negative correlations were also found after the 12-week intervention between mesomorphia and beats per minute (*p* = 0.033); LEAF-Q score and animal protein intake (*p* = 0.045); energy availability and METs (*p* < 0.001), resting caloric expenditure (*p* < 0.001) and protein intake (*p* = 0.017); and, finally, fat-free mass by bioelectrical impedance and protein intake (*p* = 0.047).

## 4. Discussion

Handball players face a variety of nutritional challenges during the competitive season. Although there has been an increase in research on nutrition and exercise over the last decade, nutrition remains a largely unknown area in sports, such as handball [47].

The main objective of this research was to evaluate the physiological and physical benefits related to RED-S of different personalized dietary plans in professional female handball players over 12 weeks. To the best of our knowledge, this study is the first to present a phenomenon of low energy availability in a group of professional female handball players. To our knowledge, there is also no research comparing the effect of different nutritional strategies on available energy, body composition, and strength performance among professional female handball players.

The prevalence of LEA was lower than 30 kcal/lean body mass per day in all female players of the team, being higher than that found in other sports, such as football, swimming, athletes, artistic gymnastics, dance, etc., (between 22 and 67%) [10,53,54]. Long-term use of such a low-energy diet can lead to serious complications, which can cause both decline in health and deterioration of exercise capacity [53]. However, accurate estimation of the prevalence of LEA remains problematic due to the variability in the methods used to estimate it.

Body composition is the parameter that most clearly improved after nutritional intervention in professional female handball players. No significant improvements were found between groups depending on the type of dietary planning followed, this may be explained by the fact that they were all correctly structured (sufficient energy, carbohydrate and protein intake) [16]. These results coincide with similar research carried out by Bellissimo et al. [55] in professional cheerleaders, where a high prevalence of LEA was also found, with a severe energy deficit and insufficient carbohydrate intake, and shown to be counterproductive in achieving optimal lean body composition. Similarly, Hooper et al. [56] found that despite athletes meeting LEA criteria and having elevated RED-S scores, the majority maintained body mass and RMR, which may explain why no significant changes were observed after the 12-week intervention, and possibly why more time may be required for changes to become evident.

Although LEA influences many body systems, such as suppression of the reproductive system and disruption of the menstrual cycle, as a mechanism to conserve energy [10], self-reported nutritional data in athletes have failed to find clear associations between LEA and menstrual disorders [7,57,58]. This is consistent with the results of the present investigation where despite the high prevalence of low energy none of the players had amenorrhea.

There is a large gap in the evidence on LEA and strength performance [10]. Only a decrease in neuromuscular performance, assessed by isokinetic dynamometry, has been observed in professional endurance athletes with menstrual dysfunction in contrast to eumenorrheic endurance athletes [59]. Although not considered significant improvements, the present intervention did show an increase in upper limb strength values over time and in CMJ jumps. This research provides valuable results on the improvement of lower limb power as assessed by the Abalakov test, where significant improvements were observed following personalized dietary-nutritional planning.

Other studies that have analyzed cardiovascular health parameters, such as blood pressure or blood cholesterol, in athletes with LEA, and also found no significant differences compared to the general population [7,60,61].

This is the first study to compare the effect of a control diet with a Mediterranean diet high in antioxidants. Although no significant differences were observed, according to the research by Schneider et al. [62], following a diet with a predominance of antioxidant foods can reduce the effects of oxidative stress caused by professional sport and improve the redox status of athletes, achieving long-term positive effects on their health. Future research should also include this variable in studies to find out how it relates to RED-S.

Several systematic reviews highlight the lack of rigorous evidence-based studies examining nutritional risks among female athletes [10,63]. Furthermore, this research responds to the need to assess the possible presence of RED-S through validated questionnaires, such as the LEAF-Q, along with assessments of biomarkers that recent reviews call for [10].

The increased prevalence of physiological and physical problems emphasizes the importance of prevention, early detection, and treatment of energy deficiency [7]. The findings suggest that interdisciplinary working groups, comprised of sports nutritionists, coaches, psychologists, and physicians, among other supportive health professionals would be beneficial for female athletes to help improve their diet, performance, and overall health [63].

To minimize limitations in this study, strict best practice protocols developed for body composition and RMR assessments were used [64,65,66,67,68]. In addition, all players comprising the professional team participated in the study, to obtain sufficient statistical power, and a 7-consecutive-day dietary recording period reflecting the typical dietary patterns of the participants was used, including heavy dietary recordings to assess energy intake. Although most of the assessments in the present study were conducted in a controlled laboratory setting, some limitations must still be acknowledged.

The first limitation is that the sample number is small, despite having all national professional female handball players of reference. In addition, another limitation is that energy expenditure was only calculated at rest, with the absence of data in the outdoors. It should be noted that measurements of body composition and menstrual cycle were not performed with gold-standard methods due to lack of material. Another limitation was due to low accessibility to material and funding, is the lack of a complete blood analysis including lipid panel, blood glucose and other values related to RED-S, such as albumin, transferrin, and antioxidant status. The lack of significant results between groups may be due to the need to follow dietary planning for longer to give markers the opportunity to show significant improvements.

The authors recognize that RED-S is a complex field for research, and the results presented in this study should be interpreted with caution, given the diversity of individual responses to intensive exercise. The lack of significant results in physiological variables and some performance test between groups may be due to the need to follow dietary planning for longer to give markers the opportunity to show significant improvements.

Future research should focus on conducting these interventions for longer and with optimal techniques, such as measuring body composition by densitometry (DXA); perform a complete analysis of biochemical parameters, such as lipidogram, glycaemia, or mineral profile; or obtaining menstrual cycle information by hormone analysis.

## 5. Conclusions

In conclusion, all professional female players who participated in the research showed low energy availability, which could increase the risk of present negative effects on physiological and physical health, that can influence sports performance. Adequate dietary plans followed for 12 weeks were sufficient to show positive results on body composition and lower limb power. However, longer interventions are needed to observe improvements in physiological variables. Future studies should be conducted in a larger group with a longer measurement period. Considering the numerous cases of low energy availability reported, it seems advisable to conduct a diagnosis of RED-S in professional team sports.

## Figures and Tables

**Table 1:** Characteristics of female handball players (mean and standard deviation).

	FD (*n* = 7)	MD (*n* = 7)	AD (*n* = 7)
Age (years)	22 ± 4	21 ± 3	22 ± 4
Height (cm)	171.0 ± 6.0	171.0 ± 7.2	173.0 ± 2.8
Weight (kg)	64.4 ± 5.1	70.5 ± 7.0	70.3 ± 6.7
BMI (kg/m^2^)	22.1 ± 1.7	24.1 ± 3.2	23.5 ± 1.9

BMI: body mass index; FD: free diet group; MD: Mediterranean diet experimental group; AD: antioxidant diet experimental group; cm: centimeters; kg: kilograms; mm: millimeters.

**Table 2:** Variables of energy availability and resting metabolic rate before, during and after the intervention in the free diet, Mediterranean diet, and antioxidant-rich diet groups.

	Free Diet (*n* = 7)						Mediterranean Diet (*n* = 7)						Antioxidant Diet (*n* = 7)						Effect Time			Effect Time × Group		
	PRE		PER		POST		PRE		PER		POST		PRE		PER		POST		F	*p*	η^2^*_p_*	F	*p*	η^2^*_p_*
	$\overset{-}{X}$	SD	$\overset{-}{X}$	SD	$\overset{-}{X}$	SD	$\overset{-}{X}$	SD	$\overset{-}{X}$	SD	$\overset{-}{X}$	SD	$\overset{-}{X}$	SD	$\overset{-}{X}$	SD	$\overset{-}{X}$	SD						
METs per minute	0.9	0.15	0.9	0.1	0.8	0.3	0.8	0.2	1.0	0.2	0.9	0.2	0.8	0.2	0.9	0.3	1.0	0.2	1.625	0.212	0.087	0.333	0.854	0.038
RMR 24 h (kcal)	1399	231	1497	184	1369	365	1349	381	1691	272	1532	318	1436	407	1619	452	1595	315	1.793	0.182	0.095	0.265	0.899	0.030
EA (kcal/day)	2.0	9.8	1.6	9.0	1.6	7.7	−2.5	10.8	−6.8	5.4	−5.3	7.1	0.8	8.0	−4.7	9.8	−1.6	7.0	1.247	0.300	0.068	0.292	0.881	0.033
LEAF-Q	8.0	1.2	9.0	2.9	9.8	3.7	9.7	3.4	9.1	2.1	8.8	2.8	9.9	3.9	10.6	3.6	10.6	3.6	1.59	0.220	0.090	1.05	0.398	0.116

EA: energy availability; MET: metabolic equivalent; RMR: resting metabolic rate; LEAF-Q: Low Energy Availability in Women Questionnaire; $\overset{-}{X}$: Mean; SD: standard deviation; PRE: before intervention; PER: during intervention; POST: after intervention.

**Table 3:** Health parameters before, during, and after the intervention of the free diet, Mediterranean diet, and antioxidant-rich diet groups.

	Free Diet (*n* = 7)						Mediterranean Diet (*n* = 7)						Antioxidant Diet (*n* = 7)						Effect Time			Effect time × Group			
	PRE		PER		POST		PRE		PER		POST		PRE		PER		POST		F	*p*	η^2^*_p_*	F	*p*	η^2^	η^2^*_p_*
	$\overset{-}{X}$	SD	$$\overset{-}{X}$$	SD	$$\overset{-}{X}$$	SD	$$\overset{-}{X}$$	SD	$$\overset{-}{X}$$	SD	$$\overset{-}{X}$$	SD	$$\overset{-}{X}$$	SD	$$\overset{-}{X}$$	SD	$$\overset{-}{X}$$	SD							
Blood pressure SYS (mmHg)	98.4	8.5	105.0	5.9	105.0	8.2	106.0	4.8	107.0	10.2	105.0	0.157	109.0	12.2	110.0	7.5	112.0	8.9	3.160	0.055	0.157	1.17	0.340	0.027	0.121
Blood pressure DIA (mmHg)	56.9	3.7	61.6	5.4	63.3	2.3	62.6	5.4	63.9	5.8	64.2	0.269	68.7	5.8	70.9	3.9	70.4	6.2	6.259	0.005	0.269	0.772	0.551	0.017	0.083
Pulse rate (bpm)	62.3	9.4	60.7	12.9	58.0	8.9	53.7	22.6	58.9	9.7	61.2	0.022	60.9	5.9	64.4	14.5	61.6	7.0	0.385	0.683	0.022	0.685	0.608	0.029	0.075
Cholesterol (mg/dL)	165.0	24.5	154.0	10.8	159.0	18.9	153.0	7.5	155.0	10.8	158.0	0.053	172.0	28.3	163.0	22.8	154.0	9.1	0.942	0.400	0.053	0.903	0.473	0.053	0.096

SYS: systolic; DIA: diastolic; mmHg: milligrams of mercury; bpm: beats per minute; mg/dL: milligrams per deciliter; $\overset{-}{X}$: Mean; SD: standard deviation; PRE: before intervention; PER: during intervention; POST: after intervention.

**Table 4:** Dietary intake variables at pre-, during, and post-intervention times in the free diet, Mediterranean diet, and antioxidant-rich diet groups.

	Free Diet (*n* = 7)						Mediterranean Diet (*n* = 7)						Antioxidant Diet (*n* = 7)						Effect Time			Effect time × Group		
	PRE		PER		POST		PRE		PER		POST		PRE		PER		POST		F	*p*	η^2^*_p_*	F	*p*	η^2^*_p_*
	$\overset{-}{X}$	SD	$\overset{-}{X}$	SD	$\overset{-}{X}$	$\overset{-}{X}$	$\overset{-}{X}$	SD	$\overset{-}{X}$	SD	$\overset{-}{X}$	SD	$\overset{-}{X}$	SD	$\overset{-}{X}$	SD	$\overset{-}{X}$	SD						
Energy intake (Kcal)	1784	373	1850	400	1740	181	1484	252	1606	163	1496	108	1820	442	1673	254	1854	329	0.026	0.975	0.002	10.265	0.408	0.108
Carbohydrates intake (%)	38.8	4.6	40.7	4.8	41.0	7.6	40.2	5.2	40.0	5.3	41.8	4.0	41.4	4.8	43.9	9.9	42.1	9.3	0.339	0.715	0.020	0.248	0.909	0.028
Protein intake (%)	20.8	3.4	22.2	3.4	20.1	2.8	21.6	4.3	21.6	2.4	21.1	2.3	21.3	1.5	22.0	3.3	22.7	3.9	1.123	0.337	0.062	0.896	0.477	0.095
Lipids intake (%)	40.3	5.2	37.2	5.5	39.2	8.1	37.8	2.5	38.0	4.5	37.2	5.0	37.4	5.2	34.2	8.0	35.0	7.9	0.995	0.380	0.055	0.197	0.938	0.023
Animal protein intake (%)	15.5	3.3	15.8	3.3	14.7	2.9	16.0	5.0	15.6	2.5	14.8	2.4	14.8	1.5	16.0	3.9	16.0	4.8	0.475	0.626	0.027	0.327	0.858	0.037
Vegetable protein intake (%)	5.3	0.83	6.2	1.0	5.3	1.0	5.5	1.0	5.9	0.9	6.1	0.5	6.5	1.0	5.7	1.0	6.7	1.6	0.248	0.782	0.014	3.374	0.020	0.284

$\overset{-}{X}$: Mean; SD: standard deviation; PRE: before intervention; PER: during intervention; POST: after intervention.

**Table 5:** Body composition variables measured by anthropometry at pre-, during and post-intervention times in the free diet, Mediterranean diet, and antioxidant-rich diet groups.

	Free Diet (*n* = 7)						Mediterranean Diet (*n* = 7)						Antioxidant Diet (*n* = 7)						Effect Time			Effect Time × Group		
	PRE		PER		POST		PRE		PER		POST		PRE		PER		POST		F	*p*	η^2^*_p_*	F	*p*	η^2^*_p_*
	$\overset{-}{X}$	SD	$\overset{-}{X}$	SD	$\overset{-}{X}$	SD	$\overset{-}{X}$	SD	$\overset{-}{X}$	SD	$\overset{-}{X}$	SD	$\overset{-}{X}$	SD	$\overset{-}{X}$	SD	$\overset{-}{X}$	SD						
Weight (kg)	64.4	5.1	65.9	4.7	65.6	5.3	70.5	7.0	70.8	5.7	69.4	5.6	70.3	6.7	70.3	7.3	70.5	7.1	3.914	0.030	0.187	0.912	0.468	0.097
6 Skinfolds (mm)	69.9	14.3	66.8	12.8	66.4	14.2	94.4	30.6	85.6	26.5	74.5	9.7	86.8	15.9	79.4	15.2	76.0	11.2	16.82	<0.001	0.497	1.29	0.292	0.132
8 Skinfolds (mm)	90.8	17.2	85.4	15.8	85.1	16.9	116.0	36.2	106.0	33.3	91.8	11.0	110.0	23.7	98.7	20.8	94.2	15.0	16.42	<0.001	0.491	1.24	0.314	0.127
Endomorphy	2.9	0.6	2.9	0.5	2.8	0.6	3.6	1.5	3.4	1.3	2.8	0.4	3.4	0.7	3.2	0.6	3.8	0.5	6.034	0.006	0.262	0.633	0.642	0.069
Mesomorphy	3.1	0.8	3.3	0.8	3.6	0.7	3.3	1.3	3.4	1.2	3.1	1.1	3.4	0.8	3.6	0.8	3.5	0.7	8.561	<0.001	0.335	0.521	0.721	0.058
Ectomorphy	2.6	1.0	2.4	0.9	2.4	1.1	1.8	1.5	1.8	1.3	2.2	0.5	2.1	0.8	2.1	0.9	2.1	0.8	3.88	0.030	0.186	1.05	0.398	0.110
Carter FM (%)	14.4	2.2	13.9	2.0	13.9	2.2	18.2	4.7	16.8	4.1	15.1	1.5	17.0	2.5	15.9	2.4	15.3	1.7	16.84	<0.001	0.498	1.29	0.293	0.132
Faulkner FM (%)	16.7	1.7	16.5	1.6	16.3	1.7	18.8	4.4	18.0	3.9	16.3	1.3	18.2	2.2	17.5	2.1	17.4	1.5	8.394	0.001	0.331	0.431	0.785	0.048
Withers FM (%)	17.6	3.3	16.3	2.1	15.6	2.7	20.9	3.1	19.3	6.2	16.9	1.1	21.1	2.7	18.4	2.8	18.5	2.1	24.13	<0.001	0.587	0.857	0.499	0.092
Carter FM (kg)	9.3	2.0	9.2	1.8	9.0	1.8	13.0	4.7	12.0	3.6	10.4	1.4	11.9	2.0	11.1	2.0	10.8	1.7	14.21	<0.001	0.455	1.34	0.274	0.136
Faulkner FM (kg)	10.8	1.8	10.7	1.7	10.5	1.7	13.4	4.4	12.8	3.9	11.2	1.3	12.8	2.0	12.3	2.0	12.2	1.6	8.765	<0.001	0.340	0.463	0.763	0.052
Withers FM (kg)	11.4	2.9	10.6	2.1	10.1	2.4	14.9	3.5	13.8	5.8	11.6	1.2	14.9	2.8	13.0	2.6	13.0	2.1	25.35	<0.001	0.599	1.02	0.412	0.107
Lee MM (kg)	25.4	2.4	22.5	2.3	22.3	2.6	25.9	2.4	22.6	2.4	22.2	2.0	26.7	3.1	23.6	3.2	23.6	3.0	198.0	<0.001	0.921	0.353	0.840	0.040
Lee MM (%)	39.4	1.9	34.1	2.3	34.0	2.8	36.9	2.4	31.9	1.9	32.1	2.2	38.0	2.1	33.5	2.3	33.4	1.8	287.3	<0.001	0.944	0.798	0.535	0.086
Rocha BM (kg)	10.1	0.92	10.1	0.9	10.1	0.9	10.2	1.0	10.2	1.0	10.3	1.0	10.5	0.5	10.5	0.5	10.5	0.5	0.895	0.418	0.050	0.921	0.463	0.098
Rocha BM (%)	15.7	1.3	15.3	1.2	15.4	1.3	14.5	1.3	14.4	1.1	14.8	0.3	15.0	1.2	15.0	1.3	14.9	1.1	3.90	0.030	0.187	1.11	0.367	0.116
RM (kg)	18.1	1.6	22.4	2.2	22.5	2.5	20.9	1.7	25.2	2.0	25.6	2.5	20.3	2.7	24.0	3.0	24.2	2.9	290.1	<0.001	0.945	0.966	0.439	0.102
RM (%)	28.1	2.2	34.0	3.2	34.3	3.1	29.8	1.9	35.7	2.9	36.8	1.5	28.8	2.1	34.0	1.6	34.3	1.3	358.6	<0.001	0.955	0.841	0.509	0.090

FM: fat mass; MM: muscle mass; BM: bone mass; RM: residual mass; kg: kilograms; mm: millimeters; $\overset{-}{X}$: Mean; SD: standard deviation; PRE: before intervention; PER: during intervention; POST: after intervention.

**Table 6:** Body composition variables measured by bioelectrical impedance at pre-, during, and post-intervention times in the free diet, Mediterranean diet, and antioxidant-rich diet groups.

	Free Diet (*n* = 7)						Mediterranean Diet (*n* = 7)						Antioxidant Diet (*n* = 7)						Effect Time			Effect Time × Group		
	PRE		PER		POST		PRE		PER		POST		PRE		PER		POST		F	*p*	η^2^*_p_*	F	*p*	η^2^*_p_*
	$\overset{-}{X}$	SD	$\overset{-}{X}$	SD	$\overset{-}{X}$	SD	$\overset{-}{X}$	SD	$\overset{-}{X}$	SD	$\overset{-}{X}$	SD	$\overset{-}{X}$	SD	$\overset{-}{X}$	SD	$\overset{-}{X}$	SD						
FF mass (kg)	49.5	3.4	50.4	4.6	49.4	3.4	50.8	3.4	52.3	1.7	51.2	3.1	53.4	5.8	52.0	5.0	52.3	5.5	0.193	0.82	0.015	2.993	0.037	0.315
FF dry mass (kg)	14.0	1.4	14.3	1.5	14.1	0.9	14.5	1.5	15.2	0.7	14.8	1.4	14.4	1.4	14.8	1.0	14.6	1.2	4.439	0.02	0.255	0.253	0.905	0.037
Lean mass (kg)	47.0	3.2	47.9	4.4	46.9	3.3	48.3	3.3	49.7	1.6	48.6	3.0	50.7	5.6	49.4	4.7	49.7	5.3	0.243	0.78	0.018	2.953	0.039	0.312
Active cell mass (kg)	29.7	2.2	30.6	3.1	29.8	1.9	30.4	2.3	31.6	1.2	30.9	2.1	31.6	3.3	31.4	2.7	31.3	3.1	2.21	0.13	0.145	1.67	0.188	0.204
Skeletal muscle mass (kg)	26.5	2.2	27.4	3.2	26.6	2.2	27.4	2.2	28.1	1.3	27.5	2.1	29.1	4.0	28.1	3.3	28.3	3.7	0.274	0.76	0.021	2.532	0.065	0.280
Metabolic protein mass (kg)	9.0	1.6	9.3	1.2	9.1	0.6	9.3	1.3	10.0	0.6	9.7	1.2	9.1	0.9	9.7	0.7	9.4	0.8	4.774	0.01	0.269	0.185	0.944	0.028
Protein mass (kg)	10.9	1.2	11.2	1.3	11.0	0.7	11.3	1.4	12.0	0.6	11.6	1.3	11.1	1.1	11.6	0.8	11.4	1.0	4.714	0.01	0.266	0.180	0.947	0.027
MM index	1.2	0.1	1.2	0.1	1.2	0.1	1.2	0.1	1.2	0.1	1.2	0.1	1.3	0.1	1.2	0.1	1.2	0.1	0.275	0.76	0.021	2.493	0.068	0.277
Constant hydration FM (%)	23.9	2.5	23.2	3.0	24.3	3.2	27.8	3.4	27.3	3.0	25.6	2.1	24.5	3.3	25.4	2.7	25.3	3.5	0.263	0.77	0.020	1.131	0.364	0.148
Gross FM (%)	24.2	2.5	23.5	3.1	24.6	3.3	28.2	3.7	27.8	3.1	26.1	2.3	24.6	3.5	25.8	2.9	25.6	3.8	0.168	0.84	0.013	1.262	0.310	0.163
FF hydration (%)	70.4	1.6	70.3	0.9	70.0	0.6	69.8	1.8	69.1	0.6	69.5	1.4	71.6	1.2	69.8	1.4	70.5	1.6	3.07	0.06	0.191	1.47	0.241	0.184
Total water (L)	35.5	2.4	36.2	3.2	35.3	2.6	36.4	2.1	37.1	1.1	36.4	1.8	39.0	4.6	37.2	4.1	37.8	4.5	0.228	0.79	0.017	3.359	0.024	0.341
Bone Mineral Content (kg)	2.5	0.2	2.5	0.2	2.5	0.2	2.6	0.2	2.6	0.9	2.6	0.2	2.7	0.2	2.6	0.2	2.6	0.2	0.869	0.43	0.063	2.264	0.090	0.258
Base metabolism (kcal)	1502	69.4	1512	75.7	1503	52.9	1518	77.2	1556	43.2	1542	73.5	1516	73.6	1536	57.7	1525	64.6	4.256	0.02	0.247	0.248	0.908	0.037
Energy expenditure (kcal)	2063	200	1921	251	1909	239	1903	141	1952	141	1973	94.1	1959	201	1928	71.6	1915	78.3	1.228	0.30	0.086	0.765	0.557	0.105

FF: fat free; FM: fat mass; MM: muscle mass; kg: kilograms; L: liters; $\overset{-}{X}$: Mean; SD: standard deviation; PRE: before intervention; PER: during intervention; POST: after intervention.

**Table 7:** Strength performance variables at pre-, during, and post-intervention times in the free diet, Mediterranean diet, and antioxidant-rich diet groups.

	Free Diet (*n* = 7)						Mediterranean Diet (*n* = 7)						Antioxidant Diet (*n* = 7)						Effect Time			Effect Time × Group		
	PRE		PER		POST		PRE		PER		POST		PRE		PER		POST		F	*p*	η^2^*_p_*	F	*p*	η^2^*_p_*
	$\overset{-}{X}$	SD	$\overset{-}{X}$	SD	$\overset{-}{X}$	SD	$\overset{-}{X}$	SD	$\overset{-}{X}$	SD	$\overset{-}{X}$	SD	$\overset{-}{X}$	SD	$\overset{-}{X}$	SD	$\overset{-}{X}$	SD						
CMJ (cm)	33.6	4.0	31.1	3.9	30.8	5.2	28.7	5.5	25.6	6.4	32.0	4.2	29.5	4.1	29.0	4.6	29.4	2.5	2.24	0.136	0.199	2.52	0.078	0.359
Abalakov (cm)	36.7	4.0	32.5	3.6	34.2	3.1	32.1	6.7	27.6	8.9	34.4	5.4	31.8	4.2	31.4	3.8	33.1	2.4	7.01	0.006	0.438	1.77	0.178	0.283
Handgrip dominant (kg)	36.8	3.1	35.5	3.2	37.2	4.1	37.1	5.2	39.0	7.6	39.3	6.6	41.9	5.6	36.3	4.3	40.0	2.0	2.891	0.077	0.208	0.962	0.448	0.149
Handgrip non-dominant (kg)	34.0	4.6	31.2	2.5	33.0	2.4	34.0	8.6	36.5	10.0	36.0	8.9	36.5	6.5	34.1	4.8	38.6	6.6	0.951	0.402	0.080	0.436	0.782	0.073

CMJ: countermovement jump; cm: centimeters; kg: kilograms; $\overset{-}{X}$: Mean; SD: standard deviation; PRE: before intervention; PER: during intervention; POST: after intervention.

## Data Availability

The data presented in this study is available on request from the corresponding author. The data are not publicly available due to personal health information.
